# Learning curve for the acquisition of 20 standard two-dimensional images in advanced perioperative transesophageal echocardiography: a prospective observational study

**DOI:** 10.1186/s12909-022-03280-3

**Published:** 2022-05-30

**Authors:** Prasert Sawasdiwipachai, Sasithorn Thanasriphakdeekul, Kasana Raksamani, Kamheang Vacharaksa, Vithaya Chaithiraphan

**Affiliations:** 1 Anesthesiology Department, Faculty of Medicine, Siriraj Hospital, Mahidol University, 2 Wanglang Road, Bangkoknoi, Bangkok, 10700 Thailand; 2 Department of Anesthesiology, Khon Kaen Hospital, 54 Sri Chant Rd, Nai Meuang, Meuang Khon Kaen District, Khon Kaen, 40000 Thailand; 3 Her Majesty Cardiac Center, Faculty of Medicine, Siriraj Hospital, Mahidol University, 2 Wanglang Road, Bangkoknoi, Bangkok, 10700 Thailand

**Keywords:** Transesophageal echocardiography, Learning curve, Cumulative success rate, Recommended caseload, Deliberate practice model, Anesthesiologists, CUSUM

## Abstract

**Background:**

Learning to perform intraoperative transesophageal echocardiography takes time and practice. We aimed to determine the cumulative success rate in the first 20 intraoperative transesophageal echocardiography cases performed by trainee anesthesiologists with no transesophageal echocardiography experience.

**Methods:**

This prospective observational study included nine anesthesiologists (four cardiovascular and thoracic anesthesia fellows and five short-course perioperative intraoperative transesophageal echocardiography trainees). Overall, 180 studies self-performed by the trainees were reviewed by certified reviewers. A study was considered successful when at least 15 qualified images were collected within 30 min. The cumulative success of each trainee was used as a surrogate of a basic two-dimensional intraoperative transesophageal echocardiography learning curve.

**Results:**

The participants comprised three male and six female anesthesiologists aged 29–43 years with 2–13 years of work experience. Most studies (146/180, 81.11%) were completed within 30 min, and the cumulative success rate was 70–90% (average 82.78 ± 6.71%). The average cumulative success rate in the short-course group (85 ± 7.07%) was higher than that in the official cardiovascular and thoracic fellow trainee group (80 ± 7.07%). The recommended caseload for a 80–100% success rate was 18–20 cases (95% confidence interval, 0.652–0.973). The CUSUM method analysis confirmed that the lower decision limit was crossed after 20 TEE studies among those achieved competence.

**Conclusions:**

We recommended a 18–20 caseload for a target success rate of 80–100% in studies performed by trainees with no previous experience. Our findings will enable the development of programs to train anesthesiologists in intraoperative transesophageal echocardiography.

**Supplementary Information:**

The online version contains supplementary material available at 10.1186/s12909-022-03280-3.

## Background

Transesophageal echocardiography (TEE) has evolved into a standard practice during open heart surgery and has become a fundamental skill that all cardiovascular and thoracic anesthesiologists must gain. Generally, TEE can be used as an intraoperative hemodynamic monitoring technique, but more specifically, it can provide significant surgery-related information, such as structural defects that mandate repair and information on the position of the cannulas or devices [1, 2].

Generally, the formal cardiovascular and thoracic fellowship training takes 1–2 years depending on the institution’s curriculum. TEE is one of the required skills among several other cardiovascular and thoracic anesthesia-related proficiencies. According to the American Society of Echocardiography/Society of Cardiovascular Anesthesiologists guideline, 20 standard two-dimensional TEE views must be part of a comprehensive examination [3]. Although new recommendations have been published [4], this guideline was used as a reference for beginners long before new technologies such as tissue Doppler and three-dimensional imaging emerged. Learning to acquire standard TEE images is a fundamental skill that should be acquired before a trainee can move on to measurements, interpretation, or more advanced TEE procedures [4].

TEE training requires basic knowledge, i.e., the principles of ultrasound, cardiac anatomy, probe manipulation, and image acquisition. This can be learned in a class or by self-study from standard textbooks. The next step involves hands-on practice. Historically, trainees would perform their first TEE examination on an actual patient (usually under anesthesia). However, currently, many simulation-based systems can facilitate such training [5–13]. To be granted a diploma in advanced perioperative TEE by the American National Board of Echocardiography, a log that includes a minimum of 150 self-performed cases and 150 TEE study reviews and reports under supervision must be submitted, in addition to passing a multiple-choice question examination [14]. To perform and interpret these many TEE studies during training, a determined effort is required. Obtaining 20 standard two-dimensional images is only the beginning of the advanced perioperative TEE training. Learning to accomplish the measurements, perform Doppler TEE, and interpret and report the results are the subsequent steps. Previous studies that reported the number of cases required by trainees have been based in intensive care unit (ICU) settings [15, 16]. With limited data regarding the learning curve for intraoperative TEE, in this study, we aimed to explore the success rate among trainees with no experience in performing intraoperative TEE.

## Methods

Following the institutional IRB approval (COA no. Si 232/2014), all participated physician trainees and involved patients provided written informed consents. The study was conducted according to the National/International/IRB guidelines. The number of required TEE studies was based on the expected final success rate of 85%. With the 95% confidence interval of 85% ± 5%, a sample of 196 studies was needed. Considering the number of cases as approximately 20–25, which are usually handled during the initial training phase in our institution in the past 5 years. As such, we planned to include 10 trainees, each performing 20 studies to a total of 200 studies for the analysis. We invited all trainees (four formal cardiovascular and thoracic fellows and six anesthesiologists) who applied for a 3-month short course on perioperative TEE training between 2014 and 2016 to participate in the study. However, one short-course TEE trainee was excluded due to her prior experience in TEE (she had performed more than 10 examinations), and a total of nine trainees were finally enrolled. All patients in this study were undergoing open heart surgery and required intraoperative TEE. They all received standard general anesthesia with an endotracheal tube. TEE probe insertion and examination began soon after anesthesia before the surgical incision was made.

All trainees received lectures on four basic topics (ultrasound principle, normal cardiac anatomy, probe placement, and image acquisition, and normal variants and artifacts). Due to the shorter training time (3 months) for the short-course trainees, they received a total of 12 h of hands-on TEE manikin simulation (HeartWorks; Inventive Medical Ltd., London, UK). All trainees also underwent a 1-h knobology session on the actual ultrasound machine (Philips iE33; Philips Ultrasound, Inc., Bothell, WA, USA). They also received one-on-one demonstrations and self-performed TEE (closely supervised) on two or three actual patients. By adopting a deliberate practice model [17–19], for their next 20 examinations, the trainees performed TEE on their own. They were instructed to digitally acquire 20 standard two-dimensional TEE images within a 30-min period that started when the first image was recorded and ended when the last image was recorded. The on-site attending anesthesiologist ensured patient safety during the examination without providing any specific guidance for image acquisition. After 30 min, the trainees could continue acquiring images, but the images acquired thereafter were not counted. All images were transferred to a server (Philips Xcelera R3.1L1, 3.1.1.422–2009). All 180 TEE studies from the nine trainees were then independently reviewed by a panel of three anesthesiologists who had passed the National Board of Echocardiography or an equivalent perioperative TEE board examination. All reviewers were blinded to the trainees’ identity, and images were accepted when each standard two-dimensional TEE image was approved by at least two out of the three reviewers. The images must contain the structure of interests in each specific view with acceptable quality, as listed in our study protocol [see Additional file 1]. The entire study was categorized as successful when at least 15 out of 20 qualified images were collected within 30 min based on data obtained from previous trainees.

### Statistical analysis

We used a binomial distribution to determine the probability of a successful or failed outcome. For the 95% confidence interval, Wald confidence intervals for *p*-values were calculated, where “p” was the success rate probability. The success rate for each attempt by each trainee was reported. We calculated the mean success rate with 95% confidence intervals of all trainees and determined the recommended mean caseload based on a success rate of 80–100% [18]. The mean and standard deviation of the success rate by the 20th TEE attempt and the time spent on each study by each trainee were calculated. Individual learning curves were constructed using the standard cumulative sum method (CUSUM) with the following parameters: probability of type I error (α) = 0.1, probability of type II error (β) = 0.1, acceptable failure rate () = 0.1 and unacceptable failure rate (p_1_) = 0.3 (Appendix A). The lower and upper decision (control) limits (h_0_ and h_1_, respectively) which correspond to the acceptable and unacceptable failure rates are then calculated based on values of α, β, p_0_ and p_1_. The individual CUSUM value starts at zero, and for each success, the amount of S is subtracted from the previous CUSUM value whereas (1-S) is added for failure. The individual CUSUM chart has the TEE study number on the horizontal axis and CUSUM value on the vertical axis. A CUSUM chart is then created by connecting each CUSUM value over the TEE study. A negative trend indicates success whereas a positive trend indicates failure. Individual CUSUM chart can be summarized by determining whether at the end of the TEE study the chart is above or below or in between the decision limits (h_1_ and h_0_). The chart that is above the upper decision limit (h_1_) at the end of the TEE study indicates that trainee’s failure rate is greater than the acceptable failure rate (no learning, no competence). On the contrary, the chart that is below the lower decision limit (h_0_) means that trainee’s failure rate is as low or lower than the acceptable failure rate (learning, competence). The chart that is between lower and upper decision limit indicates undefined performance.

Apart from the individual CUSUM charts, the average CUSUM charts from individuals with competence, without competence and having undefined performance are also constructed.

Success rate for each trainee was presented along with 95% CI. Observed success rate at each TEE study was summarized and plotted against TEE study. Calculations were performed using MS excel.

## Results

Basic demographic data of all trainees and details regarding their first 20 TEE studies are shown in Table 1. From all 180 studies, the time taken for each study ranged from 9 to 61 min with an average of 26 min. Each trainee underwent image acquisition within the 30-min timeframe, but every trainee had at least 1–9 case(s) that exceeded 30 min. The qualified images shown in Table 1 are those acquired within the permissible 30 min with acceptable quality as assessed by the panel of reviewers. The success rate of each trainee varied from 70 to 90% with the overall success rate of 82.8% (95% CI: 76.5, 88.0%, Table 2).

**Table 1:**
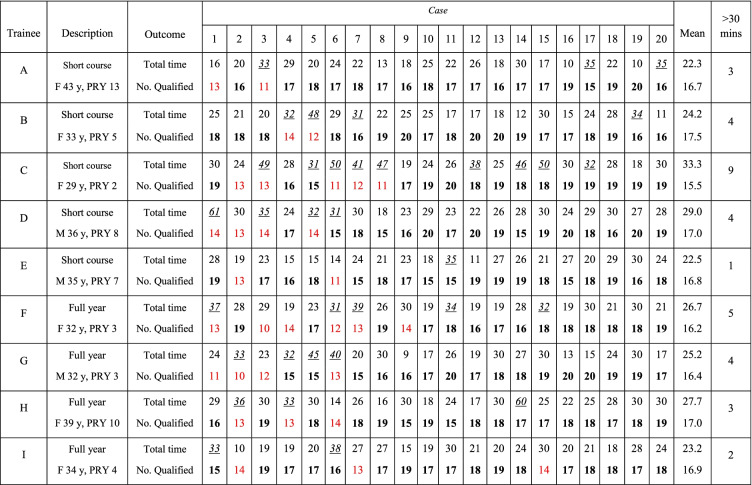
Demographic data of each trainee and details of all 20 TEE studies performed

Successful studies are indicated with bold text. The number display in red indicated the insufficient number of qualified images. Studies time longer than 30 minutes are indicated in *italic and underline*

*F* female, *y* years, *M* male, *PRY* post-residency year (number of years following completion of residency training)

**Table 2:** Individual and overall success rate out of 20 TEE studies

Trainee	Number of success	% success (95% CI)
A	18	90.0 (68.3, 98.8)
B	18	90.0 (68.3, 98.8)
C	15	75.0 (50.9, 91.3)
D	16	80.0 (56.3, 94.3)
E	18	90.0 (68.3, 98.8)
F	14	70.0 (45.7, 88.1)
G	16	80.0 (56.3, 94.3)
H	17	85.0 (62.1, 96.8)
I	17	85.0 (62.1, 96.8)
Total	149	82.8 (76.5, 88.0)

**Fig. 1:**
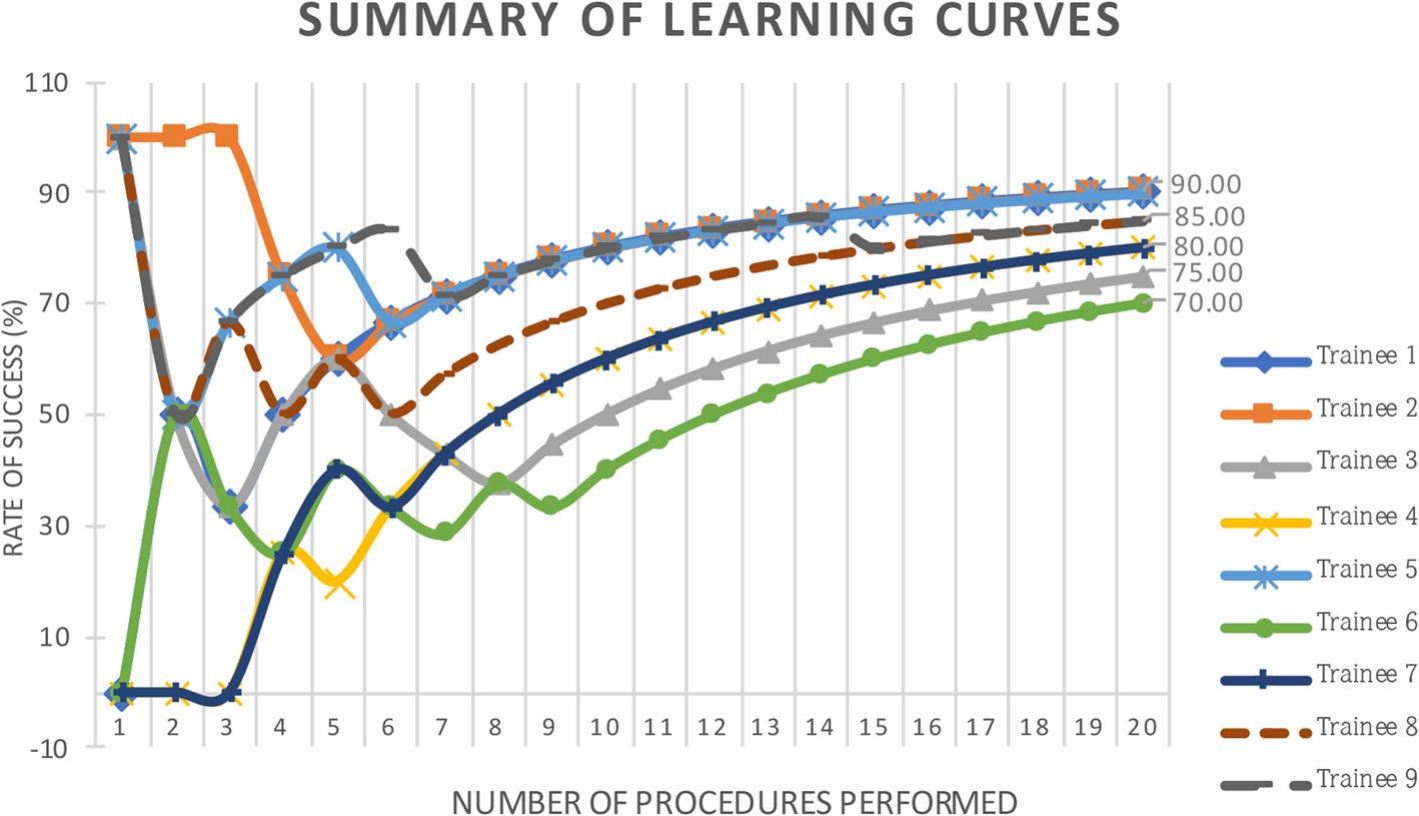
Learning curve of each trainee for performing their first 20 transesophageal echocardiography (TEE) examinations. The cumulative success rate ranges from 70 to 90% by the 20th case

There were large variations in success rate patterns among trainees, with some achieving initial success and others meeting considerably less success at the beginning (Fig. 1). However, all trainees demonstrated an increasing success rate toward their 20th case. The cumulative success rate by the 20th case was between 70 and 90% (mean 82.78 ± 6.71%). Five trainees managed to acquire all 20 standard two-dimensional TEE images (absolutely complete) within 30 min at least once in their first 20 cases. There were only nine complete studies out of the 180 examinations, representing a rate of 5%.

**Fig. 2:**
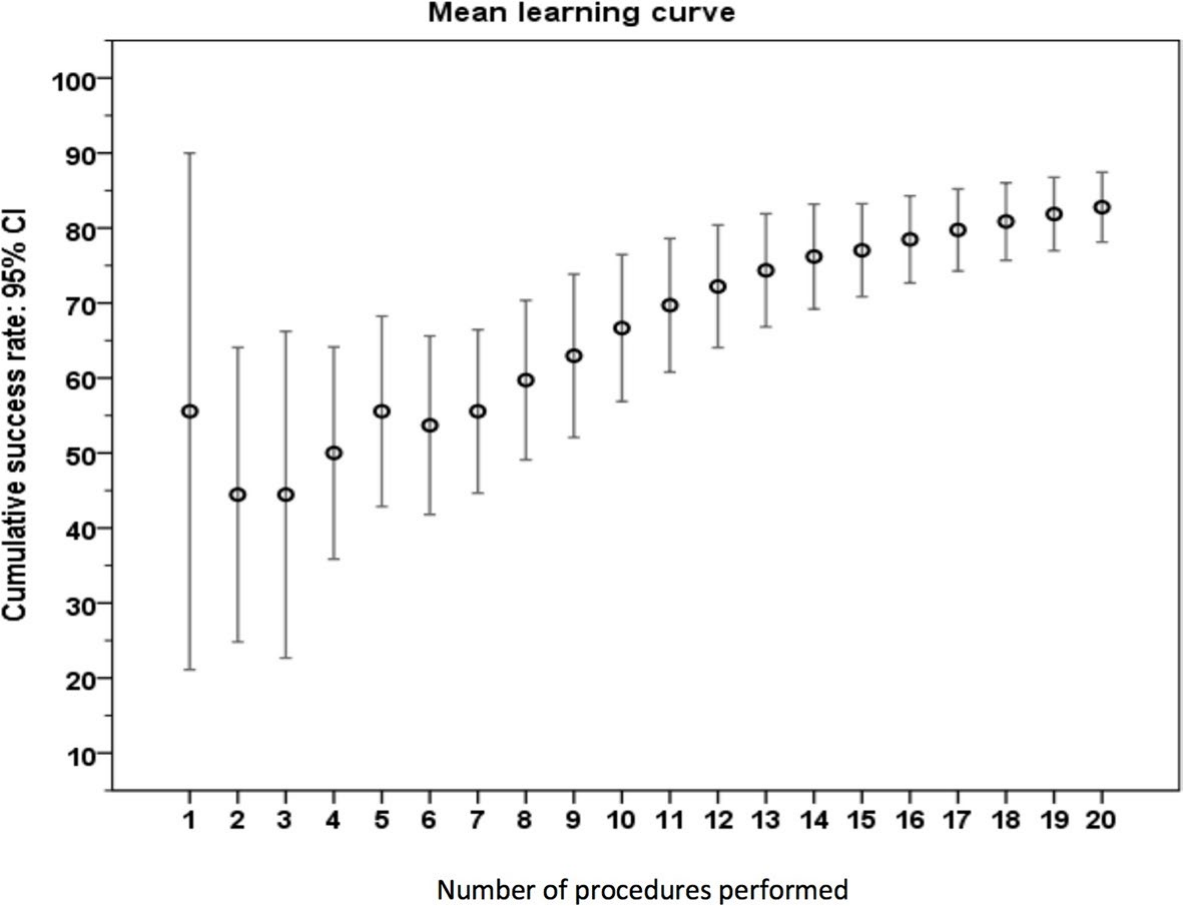
The mean learning curve of all trainees

The mean success rate of all trainees is shown in Fig. 2. Despite the early success observed in a few trainees, this was offset by some individuals with early failure. The overall pattern was an initial moderate success rate (55.56%), followed by a dip to as low as 44.44% on the second and third case, and then a gradual and steady increase up to 82.78% on the 20th case. All trainees managed to achieve a 100% success rate for their last five cases (cases 16–20).

**Fig. 3:**
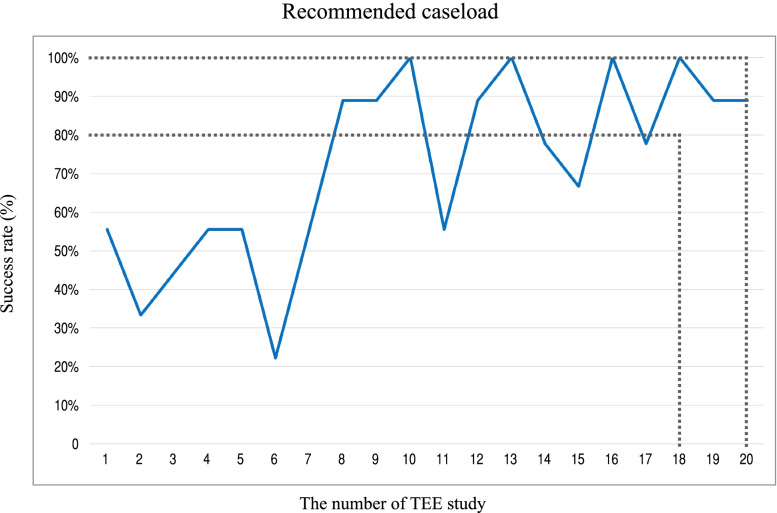
The success rate of each number of studies performed by all 9 trainees. The Y-axis represents the success rate in percents and X-axis is study number performed by all trainees

**Fig. 4:**
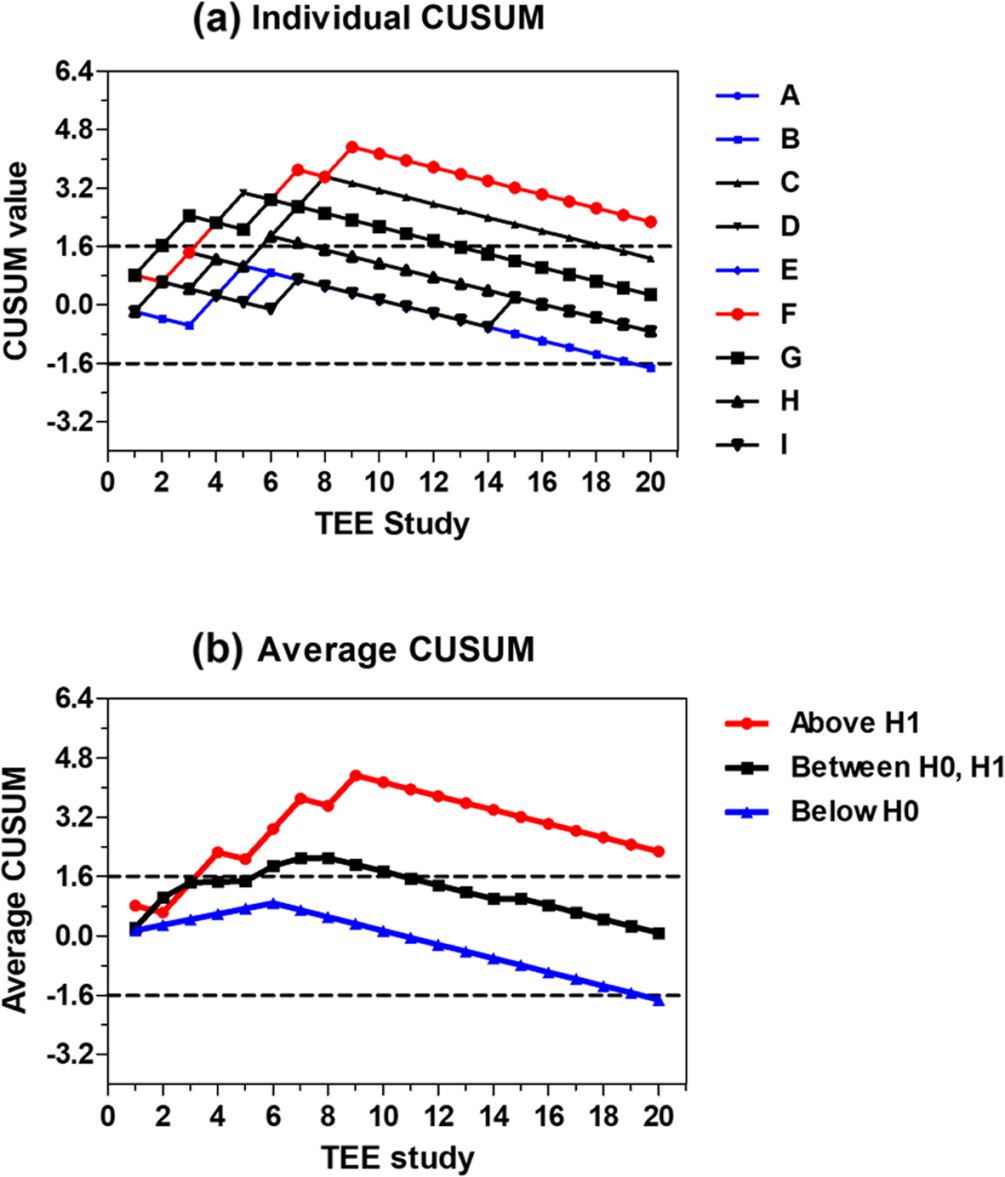
CUSUM chart for TEE study. Black dotted lines represent lower and upper decision limits of −1.6 (h_0_) and 1.6 (h_1_) respectively. Scale on the Y-axis corresponds to multiples of h_0_ and h_1_. (**a**) Individual CUSUM learning curves among 9 trainees (trainee A to I). (**b**) Average CUSUM curves for those achieving competence (blue; 3 trainees A, B and E), not achieving competence (red; 1 trainee F) and having undefined performance (black; 5 trainees C, D, G, H and I)

In terms of the recommended caseload to achieve a success rate of 80–100%, this was calculated as 18–20 cases for each trainee (Fig. 3). Based on these values, the 95% confidence interval was 0.652–0.973. The CUSUM method revealed the lower and upper decision limits of − 1.6 (h_0_) and 1.6 (h_1_) respectively. The individual CUSUM chart in Fig. 4a revealed that 3 trainees (33.3%, trainees A, B and E) achieved competence after 20 TEE studies (CUSUM is below h_0_), 1 trainee (11.1%, trainee F) did not achieve competence (CUSUM is above h_1_) and the remaining 5 trainees (55.6%, trainees C, D, G, H and I) had undefined performance (CUSUM is between h_0_ and h_1_). The average CUSUM chart among trainees who achieved competence, did not achieve competence and had undefined performance is displayed in Fig. 4b. Among those achieved competence, the lower decision limit was crossed after 20 TEE studies.

## Discussion

In this study, we determined the success rate in the first 20 intraoperative TEE procedures performed by trainees with no experience. We reported a cumulative success rate of 70–90%, with most studies completed in less than 30 min. The recommended caseload to achieve a 80–100% success rate is 18–20 cases; this only applies to the acquisition of 20 standard two-dimensional images. The CUSUM method analysis confirmed that the lower decision limit was crossed after 20 TEE studies among those achieved competence.

Learning to perform TEE has been gradually integrated into fellowship training for cardiovascular and thoracic anesthesia. Although TEE offers less degree of freedom than surface echocardiography because of the limited motion of the probe within the esophagus and stomach, manual manipulation of the TEE probe is complex and the electronic rotation of the transducer can generate an unlimited number of different imaging planes which could be overwhelming for the novice. Traditionally, learning intraoperative TEE takes considerable time, and the number of cases required to achieve an acceptable success rate has never been reported. A previous study by Charron [15] proposed that experience with 31 cases over 6 months was required, and a study by Xiang [16], only available in English as an abstract, reported that experience with 36–48 cases was required. However, both reports involved TEE performed on mechanically ventilated patients in the ICU, which is a different setting from that for intraoperative TEE.

Intraoperative TEE is often performed in anesthetized patients by a cardiovascular and thoracic anesthesiologist. In contrast, preoperative TEE is usually performed on mildly sedated patients by a cardiologist in an echocardiography suite. The main obstacles remain bright operating theater lighting, electrocautery interference, and the challenge of maintaining the balance between performing TEE and managing hemodynamics under anesthesia in patients with cardiac diseases.

It typically takes 1–2 years for a cardiovascular and thoracic fellow to develop proficiency in TEE. The National Board of Echocardiography/Society of Cardiovascular Anesthesiologists requires at least 150 self-performed TEE cases for each seeking a diploma in advanced perioperative TEE certification [14]. Each institution may have different approaches to enable their trainees to achieve this number. However, for a trainee requiring a shorter training time, a simulation-based system, e.g., HeartWorks (Inventive Medical Ltd.), can facilitate learning [5–13]. There are currently multiple TEE simulation systems, including online options, and growing evidence of the benefits of TEE teaching.

The trainees recruited for this study were within a wide range of age (29–43 years) and working experience (2–13 years). The wide variation in success rates in their initial attempts (Fig. 1) is consistent with real-life experience. In the first few attempts of novice trainees, multiple factors, such as individual predispositions (some patients may be more difficult than others), confidence, and preparation, might have a substantial impact. As more cases are completed, the influence of these factors decreases, and the trainees become more successful. This is presented in Table 1, which shows that all trainees passed their last 10 cases, except for trainee I who missed case no. 15. If we omit this single late failure, all trainees were able to succeed in their last five cases.

Most TEE studies were completed within 30 min (146/180, 81.11%). In our study, most of the prolonged studies were from a single trainee (trainee C) who accounted for 25% of the total prolonged studies (9/35). Generally, it would be difficult to perform an intraoperative TEE examination for longer than 30 min, alongside providing anesthesia care when working with an efficient surgeon. This can only happen with anesthetized patients and when the TEE examiner does not have any other responsibilities. The time could have been shortened if this trainee had been guided by an expert. Nonetheless, it is worth mentioning that more trainees will be less successful if the 30-min time limit was shortened.

This study had some limitations. First, we could not determine an appropriate method to estimate the number of trainees required for an effective sample size. The classic approach of constructing the learning curves of trainees using a cumulative sum method reported by de Oliveira Filho can be used for basic skills, such as peripheral intravenous cannulation and orotracheal intubation [20, 21]. However, TEE requires a complex skillset. With a limited number of trainees per year, we instead opted to use nQuery Advisor to determine the number of studies required. Unfortunately, our number of studies was 10% lower than expected due to exclusion of one trainee. Second, the decision to choose a trainee’s first 20 cases may appear methodologically limited. However, this number was chosen based on our site-specific data demonstrating the number of TEE cases that our trainees usually perform during the initial phase of their training (1 month for a short-course group and 3–4 months for a full-year fellow). The pace of TEE training was slower for the formal fellows, and they were required to take care of anesthetized patients while performing TEE. In contrast, the short-course trainees only performed TEE. When the overall progress of the two groups was compared, no notable differences were found. However, as this study was not designed to analyze this difference, it is inappropriate to draw any conclusions from our results. Furthermore, the CUSUM method analysis for our study has led to 5 out of 9 trainees with undefined performance despite a display of overall downward trends in Fig. 4 which is consistent with the 3 out of 9 trainees with improved performance. Thus, it is accurate to state that the outcome of 20 repetitions on these trainees is inconclusive. The herein recommended case load of 18-20 cases only applies to the acquisition of the 20 TEE images but not to their adequate interpretation which potentially would require a higher number of performed examinations. Finally, the last issue relates to the inclusion of fee-based short-course trainees in the study. However, this fee was mainly collected to ensure the institution’s procurement of equipment and consumable costs. The attending physicians involved in the study did not get paid for bedside teaching. At the time when the study was conducted, the method used was nearly identical to that of routine training. Additionally, the three panel reviewers were blind to the trainees’ identities. There was no undue influence regarding the training since the outcome of this study does not have any effects on the outcomes of the actual training.

## Conclusions

We reported a cumulative success rate of 70–90% in the first 20 cases among anesthesiologists with no previous TEE experience, which can mostly be achieved (80.55%) within 30 min. The recommended caseload to achieve a 80–100% success rate is 18–20 cases. Our findings will be useful for the development of future training programs for anesthesiologists learning to perform TEE.

## Supplementary Information

**Additional file 1.** Protocol for passing 20 standard images (additional file for use with the learning curve for transesophageal echocardiography studies only). The table describing the passing criteria used by expert reviewers for each standard TEE images. Abbreviations: PA: pulmonary artery, SVC: superior vena cava, LA: left atrium, RA: right atrium, LV: left ventricle, RV: right ventricle, RVOT: right ventricular outflow tract, IVC: inferior vena cava, LAA: left atrial appendage, LVOT: left ventricular outflow tract.

**Additional file 2.** Construction of CUSUM learning curve. The abbreviations and symbols involved in construction of CUSUM learning curve.

## Data Availability

The datasets used and/or analyzed during the current study are available from the corresponding author on reasonable request.

## Abbreviations

TEE: Transesophageal echocardiography
ICU: Intensive care unit
