# Supplementary material for: Learning curve for the acquisition of 20 standard two-dimensional images in advanced perioperative transesophageal echocardiography: a prospective observational study
Source: BMC Med Educ. 2022 May 30;22:412. doi: 10.1186/s12909-022-03280-3 (PMC9153196; doi:10.1186/s12909-022-03280-3)
Supplement: Supplementary file 1 — Additional file 1. Protocol for passing 20 standard images (additional file for use with the learning curve for transesophageal echocardiography studies only). The table describing the passing criteria used by expert reviewers for each standard TEE images. Abbreviations: PA: pulmonary artery, SVC: superior vena cava, LA: left atrium, RA: right atrium, LV: left ventricle, RV: right ventricle, RVOT: right ventricular outflow tract, IVC: inferior vena cava, LAA: left atrial appendage, LVOT: left ventricular outflow tract. [file 12909_2022_3280_MOESM1_ESM.docx]

**Additional file 1: Protocol for passing 20 standard images** (additional file for use with the learning curve for transesophageal echocardiography studies only)

|  | Probe location | Angulation | Must include | Acceptable opt out | Overall quality |
| --- | --- | --- | --- | --- | --- |
| 1 | Upper esophageal  (front) | 0° | 1. Ascending aorta 2. Right PA 3. Main PA | 1. Pulmonic valve 2. Left PA 3. SVC | ☐ OK  ☐ Borderline  ☐ Poor quality |
| 2 | Upper esophageal  (front) | 90° | 1. Ascending aorta 2. Right PA | None | ☐ OK  ☐ Borderline  ☐ Poor quality |
| 3 | Upper (mid) esophageal | 30°-60° | 1. Aortic valve (all 2 or 3 cusps) 2. LA 3. RA 4. Main PA | 1. Pulmonic valve 2. RA 3. LA 4. Inter-atrial septum | ☐ OK  ☐ Borderline  ☐ Poor quality |
| 4 | Upper (mid) esophageal | 120°-160° | 1. Aortic valve (2 cusps) 2. Aortic root 3. Sinus of Valsalva 4. Sino-tubular junction 5. Proximal ascending aorta | 1. Partial view of LA 2. Partial view of LV 3. Mitral valve 4. Partial view of ascending aorta | ☐ OK  ☐ Borderline  ☐ Poor quality |
| 5 | Mid esophageal | 0°-20° | 1. LV 2. RV 3. LA 4. RA 5. Mitral valve 6. Tricuspid valve | 1. Part of LA 2. Part of RA 3. Part of the tricuspid valve | ☐ OK  ☐ Borderline  ☐ Poor quality |
| 6 | Mid esophageal | 60°-90° | 1. RA 2. Tricuspid valve 3. RV 4. RVOT 5. Pulmonic valve | 1. Asymmetrical view of aortic valve 2. LA (partial) 3. RA (partial) 4. Main PA | ☐ OK  ☐ Borderline  ☐ Poor quality |
| 7 | Mid esophageal | 80°-110° | 1. Interatrial septum 2. LA 3. RA 4. SVC 5. IVC | 1. Partial view of LA 2. Partial view of RA 3. Partial view of SVC 4. Partial view of IVC | ☐ OK  ☐ Borderline  ☐ Poor quality |
| 8 | Mid esophageal | 60°-70° | 1. Mitral valve P3, A2, P1 2. Papillary muscle 3. LA 4. LV | 1. Partial view of LA 2. Partial view of LV | ☐ OK  ☐ Borderline  ☐ Poor quality |
| 9 | Mid esophageal | 80°-100° | 1. LAA 2. Anterior LV wall 3. Inferior LV wall 4. Mitral valve | 1. Partial LA view 2. Papillary muscle 3. Mitral leaflet (partial view) 4. Coronary sinus | ☐ OK  ☐ Borderline  ☐ Poor quality |
| 10 | Mid esophageal | 120°-160° | 1. Mitral valve 2. Aortic valve 3. Anteroseptal LV wall 4. Posterior LV wall | 1. Partial LA view 2. Partial RV view | ☐ OK  ☐ Borderline  ☐ Poor quality |
| 11 | Transgastric | 0°-20° | 1. Mitral valve (en face) 2. LV basal septal wall 3. LV basal inferior wall 4. LV basal anterior wall 5. LV basal lateral wall | 1. Partial view of basal posterior LV wall 2. Partial view of basal septal wall 3. RV | ☐ OK  ☐ Borderline  ☐ Poor quality |
| 12 | Transgastric | 0°-20° | 1. LV mid septal wall 2. LV mid anterior wall 3. LV mid inferior wall 4. LV mid-lateral wall 5. RV | 1. Partial view of mid posterior wall 2. Partial view of mid inferoseptal wall | ☐ OK  ☐ Borderline  ☐ Poor quality |
| 13 | Transgastric | 80°-100° | 1. Anterior LV wall 2. LAA 3. Mitral valve 4. Inferior wall | 1. Partial view of inferior wall 2. LV apex | ☐ OK  ☐ Borderline  ☐ Poor quality |
| 14 | Transgastric | 90°-120° | 1. Anteroseptal LV wall 2. Aortic valve 3. Posterior wall | 1. Partial view of posterior wall 2. Mitral valve 3. RV 4. LV apex | ☐ OK  ☐ Borderline  ☐ Poor quality |
| 15 | Transgastric | 100°-120° | 1. Tricuspid valve 2. RA 3. RV 4. Rt. atrial appendage | 1. RV apex 2. partial view of RA | ☐ OK  ☐ Borderline  ☐ Poor quality |
| 16 | Transgastric (deep) | 0°-20° | 1. Aortic valve 2. LVOT 3. Aortic root 4. Anterior mitral leaflet | 1. Partial view of LV 2. Partial view of RV 3. Partial view of LA | ☐ OK  ☐ Borderline  ☐ Poor quality |
| 17 | Transgastric (probe facing the back) | 0° | 1. Descending aorta (short-axis view) | 1. Pleura 2. Liver 3. Stomach | ☐ OK  ☐ Borderline  ☐ Poor quality |
| 18 | Transgastric (probe facing the back) | 90°-110° | 1. Descending aorta (long-axis view) | 1. Pleura 2. Liver 3. Stomach | ☐ OK  ☐ Borderline  ☐ Poor quality |
| 19 | Upper esophageal (probe facing the back) | 0°-15° | 1. Lower portion of ascending aorta (long-axis view) | 1. Left subclavian artery take off | ☐ OK  ☐ Borderline  ☐ Poor quality |
| 20 | Upper esophageal (probe facing the back) | 80°-110° | 1. Pulmonic valve 2. Main PA 3. Lower portion of ascending aorta (short-axis view) | 1. Left subclavian artery take off 2. Partial view of the pulmonic valve | ☐ OK  ☐ Borderline  ☐ Poor quality |

Abbreviations: PA: pulmonary artery, SVC: superior vena cava, LA: left atrium, RA: right atrium, LV: left ventricle, RV: right ventricle, RVOT: right ventricular outflow tract, IVC: inferior vena cava, LAA: left atrial appendage, LVOT: left ventricular outflow tract

-Must-include structures are prioritized; the first few structures which derive the name of the view should not be missed.

-Acceptable opt-out structures are also prioritized; the less important structures are ranked lower.

-Overall quality refers to the image quality in spatial, lateral, and temporal resolutions. The appropriate depth of field, the focus beam width, and the location should be appropriately adjusted. ECG tracing should be present. The angle of the transesophageal echocardiography plane should not be too deviated from the standard angle depicted by >20°.
