# Supplementary material for: Learning curve for the acquisition of 20 standard two-dimensional images in advanced perioperative transesophageal echocardiography: a prospective observational study
Source: BMC Med Educ. 2022 May 30;22:412. doi: 10.1186/s12909-022-03280-3 (PMC9153196; doi:10.1186/s12909-022-03280-3)
Supplement: Supplementary file 2 — Additional file 2. Construction of CUSUM learning curve. The abbreviations and symbols involved in construction of CUSUM learning curve. [file 12909_2022_3280_MOESM2_ESM.docx]

**Construction of CUSUM learning curve**

α = Probability of type I error

β = Probability of type II error

a = ln [ (1-β)/α ]

b = ln [ (1-α)/β ]

p_0_, p_1_ = Acceptable and unacceptable failure rate respectively, 0 ≤ p_0_, p_1_ ≤ 1

P = ln (p_1_/p_0_)

Q = ln [ (1-p_0_)/(1-p_1_) ]

S = Q / (P+Q)

h_0_, h_1_ = Lower and upper decision limits

h_0_ = -b / (P+Q)

h_1_ = a / (P+Q)

If α = β, then a = b and |h_0_| = h_1_ = h

CUSUM value starts at 0.

If pass (0), new CUSUM value = previous value + (0-S)

If fail (1), new CUSUM value = previous value + (1-S)
